# Supplementary material for: Direct observation of long-lived cyanide anions in superexcited states
Source: Commun Chem. 2021 Feb 10;4:13. doi: 10.1038/s42004-021-00450-0 (PMC9814559; doi:10.1038/s42004-021-00450-0)
Supplement: Supplementary file 1 — Supplementary Information [file 42004_2021_450_MOESM1_ESM.pdf]

Supplementary Information of

Direct observation of long-lived cyanide anions in superexcited states

Xiao-Fei Gao[a], Jing-Chen Xie[a], Hao Li[a], Xin Meng[a], Yong Wu[b] and Shan Xi Tian*[,a]

[a]*Hefei National Laboratory for Physical Sciences at the Microscale, Collaborative Innovation Center of Chemistry for Energy Materials (iChEM), Department of Chemical Physics, University of Science and Technology of China, Hefei 230026, China*

[b]*Institute of Applied Physics and Computational Mathematics, Beijing 100088, China*

*Corresponding author. Email: sxtian@ustc.edu.cn.

Contents

Supplementary Note 1: Vibrational states assignments

Supplementary Note 2: Angular distribution of CN^-/CN^{-}**

Supplementary Note 3: Vibrational state levels of $^3\Sigma^+$ and $^3\Pi$ states

Supplementary Note 4: Branching ratios of the CN^{-} ($^3\Sigma^+$/$^3\Pi$)**

Supplementary Note 5: Determination of the lifetime of CN^{-}**

Supplementary References

Supplementary Note 1: Vibrational states assignments

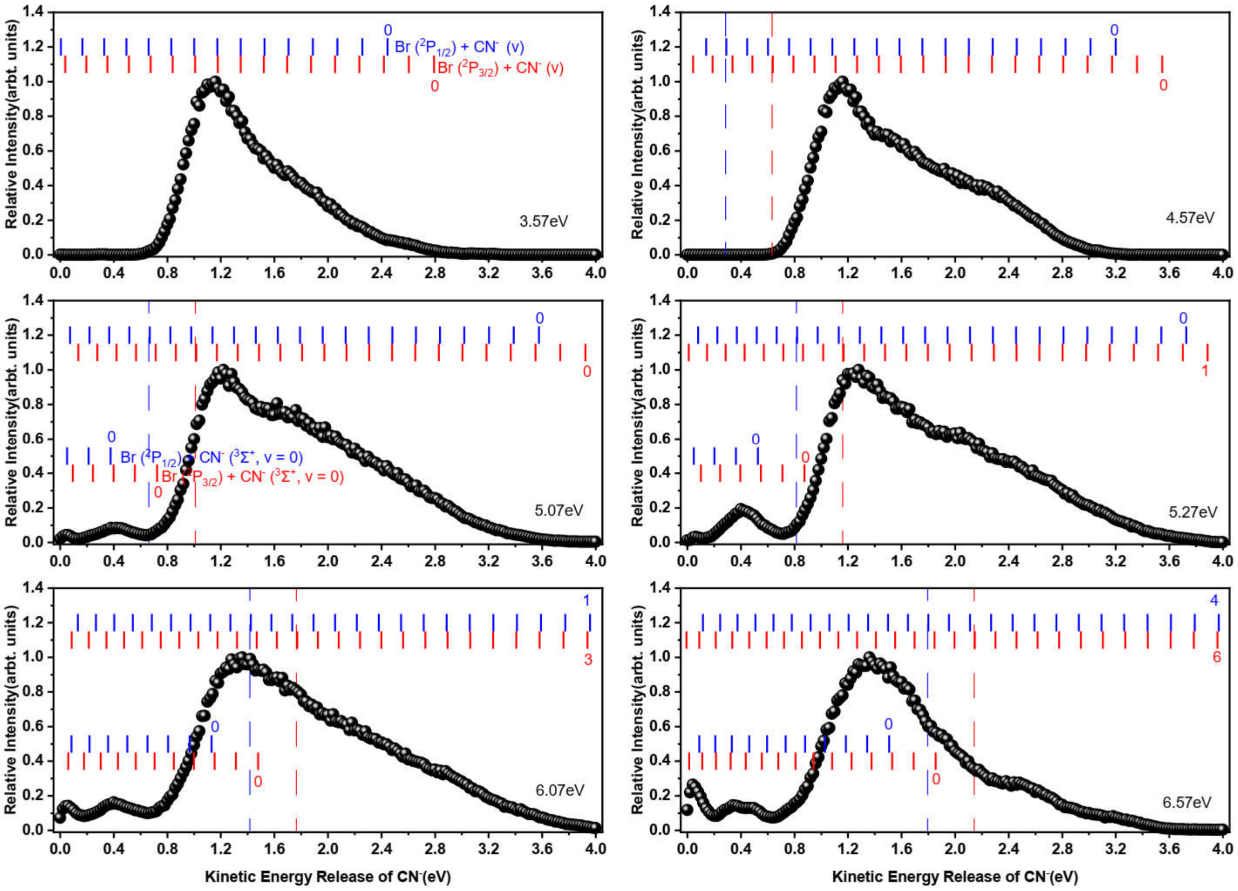

Supplementary Figure 1 | Vibrational state assignments of CN^{-} ($^{1}\Sigma^{+}$, $^{3}\Sigma^{+}$) in the kinetic energy release profiles. The intensity of each profile is normalized independently. The vertical broken lines denote the electron autodetachment thresholds of $CN^{-}(X^{1}\Sigma^{+})$ for pathways leading to $Br(^{2}P_{3/2}) + CN^{-}(X^{1}\Sigma^{+}, \nu = 0, j = 0)$ (red broken line) and $Br^{*}(^{2}P_{1/2}) + CN^{-}(X^{1}\Sigma^{+}, \nu = 0, j = 0)$ (blue broken line).

In Supplementary Figure 1, two vibrational-state serials of CN^{-} ($^{1}\Sigma^{+}$, $^{3}\Sigma^{+}$) are assigned. The vibrational levels or the kinetic energies of CN^{-} are calculated according the momentum conservation and the thresholds,

$$e^{-} + BrCN \rightarrow Br(^{2}P_{3/2}) + CN^{-}(X^{1}\Sigma^{+}, \nu = 0, j = 0) \quad E_{th} = -0.13 \text{ eV}$$

$$\rightarrow \quad Br^{*}(^{2}P_{1/2}) + CN^{-}(X^{1}\Sigma^{+}, \nu = 0, j = 0) \; E_{th} = 0.33 \text{ eV}$$

where only isotopic atom ^{79}Br is considered. More details about the state assignments can be found in Supplementary Note 3. The small mass difference between ^{79}Br and ^{81}Br leads to a difference about 0.5% of the CN^{-} kinetic energy. Therefore, we ignore this isotope effect in the following discussion.

Besides the superexcited bound states of CN^{-}, the electronically excited states ($^{4}P_{5/2,3/2}$) of the co-product Br are also possibly presented but their related pathways (the thresholds are given in the following) are not accessible in the electron attachment energy range of 3.57 - 6.57 eV.

$$e^{-} + BrCN \rightarrow Br^{*}(^{4}P_{5/2}) + CN^{-}(X^{1}\Sigma^{+}, \nu = 0, j = 0)\ E_{th} = 7.73\ eV$$

$$\rightarrow Br^{*}(^{4}P_{3/2}) + CN^{-}(X^{1}\Sigma^{+}, \nu = 0, j = 0)\ E_{th} = 7.92\ eV$$

Supplementary Note 2: Angular distribution of CN^{-}/CN^{-}**

As discussed in the main text, detailed information about the resonant states of $BrCN^{-}$ formed in the present collision energy range and their full-dimensional potential energy surfaces is required to elucidate the angular distributions of the cyanide anionic products. They are still in lack, although we have successfully evaluated the potential energy surfaces at the low-lying states of $BrCN^{-}$ predicted with the high-level calculations (supplementary ref. 1). Here, we present an analysis about the angular distributions of CN^{-}/ CN^{-}** recorded at 6.07 eV (as an example) in a straight way. As shown in Supplementary Figure 2, the maximum intensity for $CN^{-}(X^{1}\Sigma^{+}, \nu < 17)$ locates around the scattering angle of 110°, about 0° for CN^{-}** $(X^{1}\Sigma^{+}, \nu \geq 18)$ and 95° for CN^{-}** $(^{3}\Sigma^{+}/^{3}\Pi)$. The distinct difference of between the formers should arise from two different DEA pathways. Sophisticated theoretical calculations are demanded to have more insights into their dynamics.

The intersystem transition from the superexcited vibrational states of $X^{1}\Sigma^{+}$ to $^{3}\Sigma^{+}$ happens for the CN^{-} yield which has been produced in the DEA process, thus the angular distribution of CN^{-}** $(X^{1}\Sigma^{+}, \nu \geq 18)$ is directly determined with the DEA dynamics, rather than the post-dissociation intersystem transition. On the other hand, the bromine isotopic effect is too weak to influence the CN^{-} / CN^{-}** angular distributions.

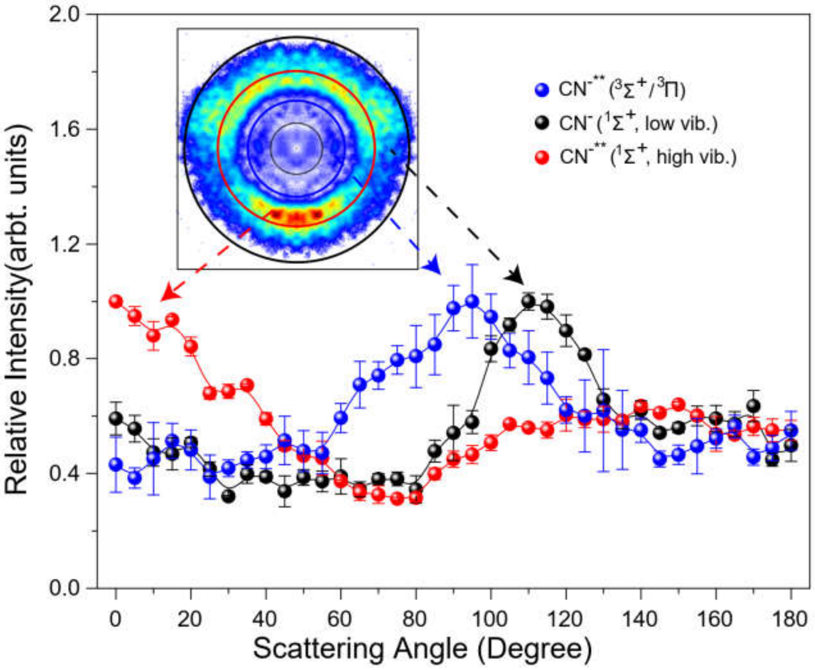

Supplementary Figure 2 | Angular distributions of CN^{-}/CN^{-} at the collision energy of 6.07 eV.** The intensity of each profile is obtained by summarizing all signals in the corresponding annular and then normalized

independently. CN^-($X^1\Sigma^+$, ν < 17) and CN^{-}** ($X^1\Sigma^+$, ν ≥ 18) define the low and high vibrational-state contributions, respectively.

Supplementary Note 3: Vibrational state levels of $^3\Sigma^+$ and $^3\Pi$ states

We repeated the calculations to obtain the potential energy curves at the same level of theory (icMRCI + CASSCF)[2]. In the present calculations with MOLPRO suite of programs[3], the aug-cc-pVQZ basis set[4] was supplemented with a set of diffuse s-type functions, namely, with the parameters of 1.121000E-2 for C and 3.530000E-2 for N. In comparison with the previous results[5], the present curves, as shown in Figure 4, are smoother, and no singularities are presented.

The electron configuration of CN($X^2\Sigma^+$) is $(1\sigma)^2(2\sigma)^2(3\sigma)^2(4\sigma)^2(1\pi)^2(2\pi)^2(5\sigma)^1(6\sigma)^0$, while those of CN^- are:

$$^1\Sigma^+: (1\sigma)^2(2\sigma)^2(3\sigma)^2(4\sigma)^2(1\pi)^2(2\pi)^2(5\sigma)^2(6\sigma)^0,$$

$$^3\Sigma^+: (1\sigma)^2(2\sigma)^2(3\sigma)^2(4\sigma)^2(1\pi)^2(2\pi)^2(5\sigma)^1(6\sigma)^1,$$

$$^3\Pi: (1\sigma)^2(2\sigma)^2(3\sigma)^2(4\sigma)^2(1\pi)^2(2\pi)^{1\uparrow}(5\sigma)^2(6\sigma)^{1\uparrow},$$

$$^1\Pi: (1\sigma)^2(2\sigma)^2(3\sigma)^2(4\sigma)^2(1\pi)^2(2\pi)^{1\downarrow}(5\sigma)^2(6\sigma)^{1\uparrow},$$

where the electron spins of 2π and 6σ are parallel in $^3\Pi$ state and antiparallel in $^1\Pi$ state.

As shown in Figure 4, all of above superexcited states are bound states. We derive their vibrational-state energy levels, by fitting the calculated potential energy curves with the ninth-order polynomials. Then the molecular constants (like w_e and w_ex_e), zero-point energy (ZPE) and energies of CN and CN^- are obtained and list in Supplementary Table 1.

Supplementary Table 1 | Energy position (E), zero-point energy (ZPE) and molecular constants of CN^- and CN

		E(ν=0, j=0) /eV	ZPE/eV	w_e/eV	w_ex_e/eV
CN^-	$X^1\Sigma^+$	0 [a]	0.1256	0.2519	0.0014
CN^-**	$^3\Sigma^+$	4.2415	0.1118	0.2254	0.0034
	$^1\Pi$	6.3830	0.1167	0.2354	0.0037
	$^3\Pi$	5.8166	0.0784	0.1576	0.0022
CN	$^2\Sigma$	3.8270	0.1243	0.2494	0.0015

[a] The reference energy.

Using the parameters in Supplementary Table 1 and the following formula,

$$E_v = E + w_e\left(v + \frac{1}{2}\right) - w_ex_e\left(v + \frac{1}{2}\right)^2 \quad \text{(Supplementary Equation 1)}$$

We further obtain the vibrational levels of CN^{-}/CN^{-**}, and the results are listed in Supplementary Table 2.

Supplementary Table 2 | Vibrational levels of CN^{-}/ CN^{-**}

v	E_v/eV[a]			
	$X^1\Sigma^+$	$^3\Sigma^+$	$^1\Pi$	$^3\Pi$
0	0.1256	4.3918	6.5333	5.9669
1	0.3747	4.6104	6.7613	6.1201
2	0.6210	4.8222	6.9819	6.2689
3	0.8645	5.0272	7.1951	6.4133
4	1.1052	5.2254	7.4009	6.5533
5	1.3431	5.4168	7.5993	6.6889
6	1.5782	5.6014	7.7903	6.8201
7	1.8105	5.7792	7.9739	6.9469
8	2.0400	5.9502	8.1501	7.0693
9	2.2667	6.1144		7.1873
10	2.4906	6.2718		7.3009
11	2.7117	6.4224		
12	2.9300	6.5662		
13	3.1455	6.7032		
14	3.3582	6.8334		
15	3.5681	6.9568		
16	3.7752	7.0734		
17	3.9795			
18	4.1810			
19	4.3797			
20	4.5756			
21	4.7687			
22	4.9590			
23	5.1465			
24	5.3312			
25	5.5131			
26	5.6922			
27	5.8685			
28	6.0420			
29	6.2127			
30	6.3806			
31	6.5457			
32	6.7080			
33	6.7419			

[a] The energy values are respective to the minimum of the potential energy curve of $CN^{-}(X^1\Sigma^+)$.

Supplementary Note 4: Branching ratios of the CN^{-} ($^3\Sigma^+/^3\Pi$)**

As shown in Figures 3c-3f, the small peaks at the left side are attributed to the CN^{-}** ($^3\Sigma^+/^3\Pi$) yields that are directly produced in the DEA processes, instead of those formed via the intersystem transition from the superexcited vibrational states of the free fragment CN^{-}**($X^1\Sigma^+$). Thereby, we can derive their branching ratios in terms of the electron attachment energy. As shown in Supplementary Figure 3, the direct productions of this metastable CN^{-}** are enhanced with the increase of attachment energy.

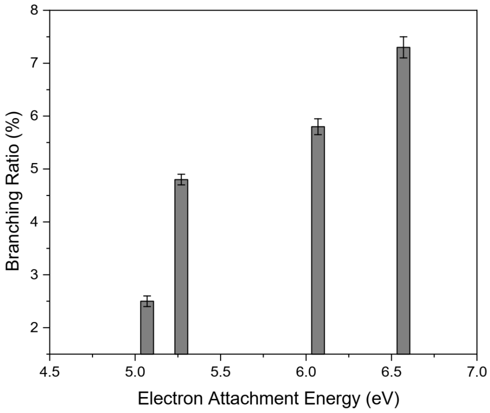

Supplementary Figure 3 | Branching ratios of the CN^{-} ($^3\Sigma^+/^3\Pi$).** The ratio is given as the CN^{-}** ($^3\Sigma^+/^3\Pi$) proportion of the total CN^{-} yields at each electron attachment energy.

Supplementary Note 5: Determination of the lifetime of CN^{-}**

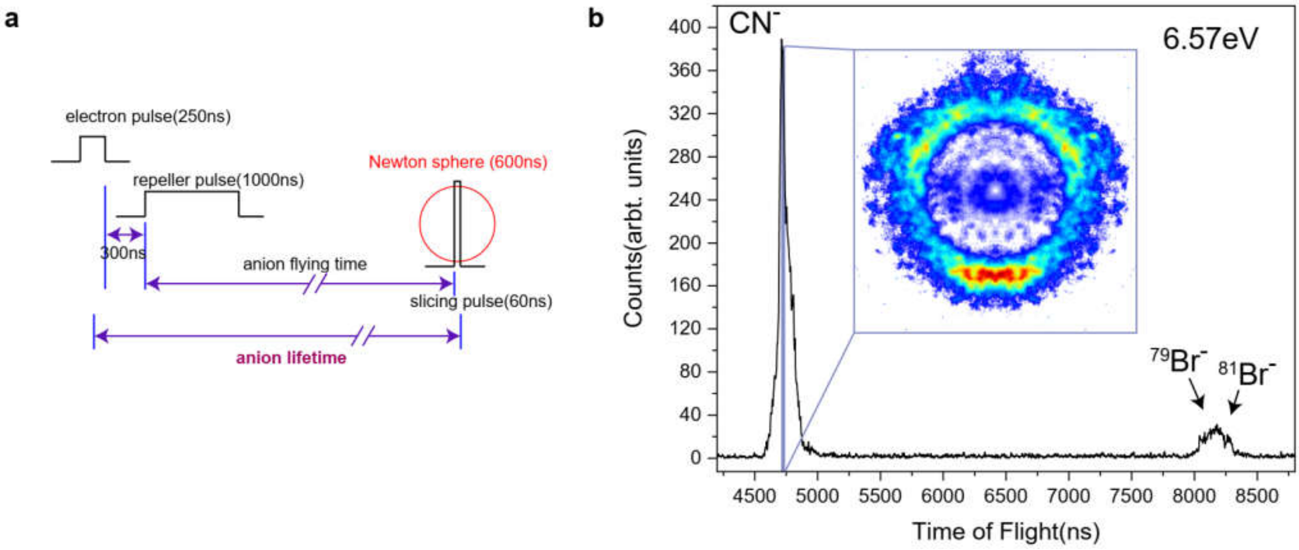

Supplementary Figure 4 | Time sequence of the pulses used in the measurements (a) and a mass spectrum of anionic products at the collision energy of 6.57 eV. a The lifetime of anionic fragment is not less than its flying time plus the delaying time between the electron and repeller pulse. The slicing pulse width (60 ns) is a tenth of the time diameter of Newton sphere. **b** Besides the CN^{-} products, the isotopic products $^{79}Br^{-}$ and $^{81}Br^{-}$ are observed. A

vertical bar as a central slice of the CN^{-} peak is selected for the velocity imaging measurement and the time-sliced velocity image is shown as an inset picture.

As shown in Supplementary Figure 4a, the repeller pulse is delayed with 300 ns to remove the residual electrons after the DEA reactions. The reason of this operation has been elaborated[6]. The time diameter of the Newton sphere of CN^{-} products is about 600 ns (see the bottom width of the CN^{-} peak in Supplementary Figure 4b), and the time-slicing thickness is 60 ns. Therefore, except for the CN^{-} ($X^1\Sigma^+$, $\nu \leq 17$), the CN^{-}** lifetime is not less than a sum of the flying time (about 4700 ns) and the delaying time (300 ns, between the electron and repeller pulse), namely, about 5000 ns.

Supplementary References

(1) X. F. Gao, F. An, H. Li, J.-C. Xie, X.-D. Wang, X. Meng, B. Wu, D. Xie & S. X. Tian, Probing the potential energy surfaces of $BrCN^{-}$ by dissociative electron attachment. *J. Phys. Chem. Lett.* **11**, 9110-9116 (2020).

(2) H.-J. Werner & P. J. Knowles, An efficient internally contracted multiconfiguration-reference configuration interaction method. *J. Chem. Phys.* **89**, 5803-5814 (1988).

(3) H.-J. Werner, P. J. Knowles, G. Knizia, F. R. Manby & M. Schütz, Molpro: a general-purpose quantum chemistry program package. *WIREs Comput. Mol. Sci.* **2**, 242-253 (2012).

(4) R. A. Kendall, T. H. Dunning, Jr. & R. J. Harrison, Electron affinities of the first-row atoms revisited. Systematic basis sets and wave functions. *J. Chem. Phys.* **96**, 6796-6806 (1992).

(5) R. Polák & J. Fišer, On the electronic structure of CN^{-}. *J. Mol. Struct. (Theochem)* **584**, 69-77 (2002).

(6) X.-F. Gao, H. Li, X. Meng & S. X. Tian, Ion-pair dissociations of BrCN by electron impacts. *Chin. J. Chem. Phys.* **32**, 89-92 (2019).
